# Successful Therapy over 12 Months of People with Cystic Fibrosis with Rare Non-phe508del Cystic Fibrosis Transmembrane Conductance Regulator (CFTR) Mutations with Elexacaftor/Tezacaftor/Ivacaftor (ETI)

**DOI:** 10.3390/arm92060049

**Published:** 2024-12-20

**Authors:** Tomke Sütering, Sebastian F. N. Bode, Rainald Fischer, Dorit Fabricius

**Affiliations:** 1 Department of Pediatrics and Adolescent Medicine, Ulm University Medical Center, Ulm University, 89075 Ulm, Germany; tomke.suetering@uni-ulm.de (T.S.); sebastian.bode@uniklinik-ulm.de (S.F.N.B.); 2 Cystic Fibrosis Center Munich West, Ludwig Maximilian University (LMU), 81241 Munich, Germany; rainald.fischer@gmail.com

**Keywords:** elexacaftor/tezacaftor/ivacaftor, cystic fibrosis, rare CFTR mutations, non-phe508del CFTR mutations, off-label use

## Abstract

**Highlights:**

**What are the main findings?**

- CFTR modulator therapy Elexacaftor/Tezacaftor/Ivacaftor (ETI) is effective in people with CF (pwCF) with rare non-phe508del CFTR mutations that permit the production of some CFTR protein.
- Beneficial clinical ETI effects such as improved lung function, reduced symptoms, and enhanced quality of life remained stable over an observation period of 12 months.

**What is the implication of the main finding?**

- In this report, it is suggested that Elexacaftor/Tezacaftor/Ivacaftor (ETI) can be effective for pwCF who have rare, ETI-responsive non-phe508del mutations, potentially supporting the expansion of its approved use in Europe, based on in vitro responsiveness.

**Abstract:**

**Background:** Elexacaftor/Tezacaftor/Ivacaftor (ETI) is a CFTR modulator therapy approved for people with cystic fibrosis (pwCF) who have at least one phe508del mutation. However, its approval in the European Union (EU) for pwCF with non-phe508del mutations is lacking, because data on treatment response in this subgroup are scarce. **Methods:** This retrospective observational study evaluated six pwCF (ages 6 to 66) with responsive CFTR mutations (M1101K, R347P, 2789+5G>A, G551D) undergoing off-label ETI therapy. Evaluations were conducted at 0, 3, 6, 9, and 12 months, assessing lung function (FEV_1_), sweat chloride levels, body mass index (BMI), quality of life, medication satisfaction, ear, nose and throat (ENT) symptoms, and physical activity. A control group of four pwCF with classic symptoms and no ETI treatment was included. **Results:** FEV_1_ improved significantly after 3 and 6 months (*p* < 0.05) and stabilized by 12 months. Sweat chloride levels decreased significantly, with four pwCF achieving levels <60 mmol/L. Improvements in the upper and lower airway symptoms, medication satisfaction, and increased BMI were noted. **Conclusions:** ETI demonstrates high efficacy in this small group of pwCF with rare CFTR mutations, offering a treatment option that warrants further monitoring and evaluation.

## 1. Introduction

Cystic fibrosis (CF) is an autosomal recessive hereditary disease that affects over 105,000 people worldwide. High prevalence is mainly found in the Caucasian population in the northern hemisphere, with recently published studies suggesting an unreported figure of over 50,000 in other parts of the world [1]. CF is caused by mutations in the gene encoding the cystic fibrosis transmembrane conductance regulator (CFTR) channel protein, located in the secretory glandular epithelium of multiple organ systems such as the airways, sweat glands, gastrointestinal tract, hepatobiliary system, reproductive system, and pancreas, where it plays an important role in epithelial bicarbonate and chloride transport. More precisely, the disturbed metabolism causes a pathologically composed glandular secretion, the removal of which is disturbed by increased viscosity. CF is therefore characterized by pulmonary obstruction, pancreatic insufficiency, reduced fertility, and an increased salt content in sweat [2]. Currently, people with CF (pwCF) in Germany still have a reduced life expectancy, due to cardiopulmonary causes, diabetes, and liver failure [3]. As a chronic and life-shortening illness, CF leads to twice the prevalence of depression and anxiety disorders in pwCF and their caregivers compared to the normal population [4].

CFTR modulators are small molecules that intervene directly in the defective CFTR protein production and can also improve the function of the CFTR channels [5]. Since 2020, the CFTR modulator Elexacaftor/Tezacaftor/Ivacaftor (ETI) has been the first highly efficient causal therapeutic approach available for the vast majority of pwCF in the European Union (EU). More specifically, ETI is currently authorized by the European Medicines Agency (EMA) for all pwCF aged 2 years and older with at least one phe508del CFTR mutation [6], but not for pwCF with much rarer non-phe508del mutations.

Clinically, the effect of CFTR modulators is primarily seen in an improvement in lung function (forced expiratory volume in 1 s, FEV_1_), sweat chloride levels, body mass index (BMI), and a reduction in exacerbations [6,7]. Recent studies evaluated over 2600 pwCF in Germany for 24 weeks on ETI therapy, confirming the positive effects of ETI on lung function and sweat chloride concentration [8]. Moreover, the validated Cystic Fibrosis Questionnaire-Revised (CFQ-R), a disease-specific health-related quality of life measure for children, adolescents, and adults with cystic fibrosis (CF) was included in the evaluation of ETI effects [8]. Since the introduction of ETI, the life expectancy of newborn pwCF in Germany has increased from 53 years in 2019 to 60 years in 2022 [3,9].

In addition to the classic improvements in FEV_1_, BMI, and sweat chloride concentration, improvements in physical resilience [10], or the condition of the upper airways have now also been shown. The improved ear, nose and throat (ENT) symptoms under ETI have been demonstrated in several studies through significantly better scores in the Sinonasal Outcome Test 22 (SNOT-22) questionnaire [11,12,13], by radiological imaging [14], as well as through objective examinations by an otorhinolaryngologist [15]. Even though there are some case reports on the occurrence of ETI-associated depression and anxiety [16,17], a recently published review considering over 14 clinical trials and over 60,000 pwCF on ETI could not prove a causal relationship between ETI and depression, and a prospective study from 2023 even reported a significant decrease in depressive symptoms during ETI therapy [18,19].

In contrast to the EU, the ETI authorization in the USA was extended by the Food and Drug Administration (FDA) to include 177 rarer non-phe508del CFTR mutations, which were not necessarily responsive to Ivacaftor or Tezacaftor/Ivacaftor [6], but for which the ETI-producing pharmaceutical company had provided unpublished in vitro data, demonstrating some CFTR function, regardless of the mutation class or the underlying mechanism, therefore defined as “responsive” to ETI [20]. This was based on the following in vitro data: CFTR wild-type activity was measured in Fisher rat thyroid (FRT) cell cultures transduced with various CFTR mutations after the addition of ETI. ETI was considered effective for a certain CFTR mutation if chloride transport increased by at least 10% of normal (wild-type chloride transport) compared to baseline [21], as the threshold for CF diagnosis was less than 10% CFTR wild-type activity [22]. Based on this decision, the authorization for this pwCF subgroup has also been extended in the UK.

As authorizations based on in vitro data are not permitted in the EU and the pwCF subgroup with rare non-phe508del CFTR mutations is too small for a pivotal trial, extension of ETI authorization in the EU will not be feasible in the foreseeable future. Therefore, we have started to prescribe ETI off-label for pwCF with FDA-approved non-phe508del-responsive CFTR mutations following a successful application to the health insurance providers. Apart from data from the French Compassionate Use Program (FCUP) published at the beginning of 2023 [23] and a few individual case reports [24,25,26], there are hardly any data worldwide and none in Germany on the treatment response of ETI in pwCF with non-phe508del CFTR mutations, even though some responsive CFTR mutations are included in the current study VX21-121-445, a phase 3 trial evaluating ETI effects in pwCF 6 years and older with non-phe508del CFTR mutations, with so far unpublished results.

## 2. Materials and Methods

### 2.1. Study Design

This retrospective two-center, longitudinal study was considered as an evaluation of case reports. Since the start of CFTR modulator therapy, the centers at Ulm University Hospital (UUH) and Munich Pasing Lung Clinic decided to include questionnaires as outlined below in our routine care. The data were collected at routine appointments in the period from February 2021 to February 2024. All pwCF in the two centers have three-monthly outpatient appointments. These appointments include pulmonary function tests, laboratory chemistry results, a detailed medical history, and a clinical examination. Sweat chloride was measured before and at least three months after starting treatment with a new CFTR modulator. In addition, the following questionnaires were used: SNOT-22, CFQ-R, and a self-designed Activity Questionnaire (AQ), as well as Treatment Satisfaction Questionnaire for Medication (TSQM). No data deviating from the routine were collected. All data were analyzed retrospectively after the end of the survey period.

Six pwCF aged 6 to 66 years (3 female, 3 male) with at least one FDA-approved non-phe508del CFTR mutation from the Cystic Fibrosis Outpatient Clinic at UUH and the Munich Pasing Lung Clinic were evaluated immediately before and 3/6/9/12 months (+/−1) after initiation of ETI therapy. ETI was prescribed off-label in the dosage recommended by the manufacturer after approval of reimbursement by the health insurance companies. Four pwCF without ETI therapy with classic CF symptoms served as a control group. Exclusion criteria were organ transplants, acute infections, unstable pulmonary or cardiovascular disease, pregnancy, recent pneumothorax (<6 weeks), relevant hemoptysis (old blood > 5 mL or fresh blood more than trace < 4 weeks), pronounced liver disease (cirrhosis with portal hypertension, Child Pugh Score > 7), and malignant disease.

Clinical parameters such as lung function, sweat test, BMI (percentile in children), and laboratory values were observed. Lung function testing was performed using body plethysmography (Vyaire Master Screen Body with Vyaire software SentrySuite, version 3.10.3), according to Global Lung Function Initiative/European Respiratory Society guidelines. In addition, the development of ENT symptoms was assessed with the SNOT-22 questionnaire [27], quality of life with the CFQ-R [28], and medication satisfaction with the TSQM [29]. Everyday activity and physical performance were measured using a self-designed Activity Questionnaire (AQ), which contained individual elements of the BSA questionnaire (German: Bewegungs- und Sportaktivität, BSA, English: Motility and Sports activity), for measuring physical activity [30]. The current number of minutes of walking and cycling without a break and the number of stairs that could be climbed were queried. In addition, the average number of steps per day in the week of the outpatient appointment was queried if a pedometer, including a smartphone that can count steps, was available.

### 2.2. Data Analysis and Statistics

All data sets were collected using Microsoft Excel (Microsoft Corporation, Redmond, WA 98052-6399, USA), and the corresponding files were stored on a secure, access- and password-restricted server, based at Ulm University Hospital. Descriptive statistics to analyze the patient collectives, individual values, minima, maxima, median, and interquartile ranges. Statistical analyses were performed with Graph Pad Prism (Version 10.1.0., GraphPadSoftware, La Jolla, CA, USA, www.graphpad.com, accessed on 26 April 2024). Due to the small number of patients, only non-parametric tests were used. The significance level was set at *p* < 0.05 in all analyses. The non-parametric Friedman test was performed to test for significant differences in the parameters at more than two different time points after the start of ETI therapy. The non-parametric Wilcoxon matched-pairs signed rank-test was performed to test for differences before and during ETI therapy at one certain time point, the Wilcoxon–Mann–Whitney test was used to test for significant differences between the target group and the control group at the same time point.

### 2.3. Ethics

An ethics vote was waived by the Ethics Committee of Ulm University, as this is a retrospective study that is considered as an evaluation of case reports (Chairman Florian Steger; No. 114/23, approved 24 May 2023). This study was conducted in accordance with the Declaration of Helsinki. Patients were informed about this study by their treating physicians and by written information material. They had the opportunity to ask questions at any time. PwCF were included in this study after they had given their written informed consent. None of the patients withdrew their consent at any time during this study.

## 3. Results

### 3.1. Cohort

The study included 6 pwCF (3 male, 3 female) aged 6 to 66 years who carried at least one FDA-approved non-phe508del CFTR mutation with presumed responsive CFTR function, sometimes in combination with nonsense mutations. Patient 6 had already received another CFTR modulator (Ivacaftor) prior to starting ETI therapy. Detailed demographic data for the intervention and control group can be found in Table 1.

The pwCF with CFTR mutations R347P/R1066C who underwent ETI therapy was evaluated at time points 0, 3, and 6 months, but had to be excluded from the time points 9 and 12 months due to the occurrence of a malignant disease and the associated fulfillment of the exclusion criteria. The data of this pwCF are still included at time points 0, 3, and 6 months. Further observational data on this patient undergoing ETI are highlighted in gray in the tables and graphs. A total of four pwCF (three female/one male) with classic symptoms without ETI therapy aged between 7 and 44 years (with the mutations Q39X, R785X, phe508del, I506S, CFTRdele17a,17b, CFTRdele14b-17b) served as a control group.

### 3.2. Clinical Parameters

**Figure 1:**
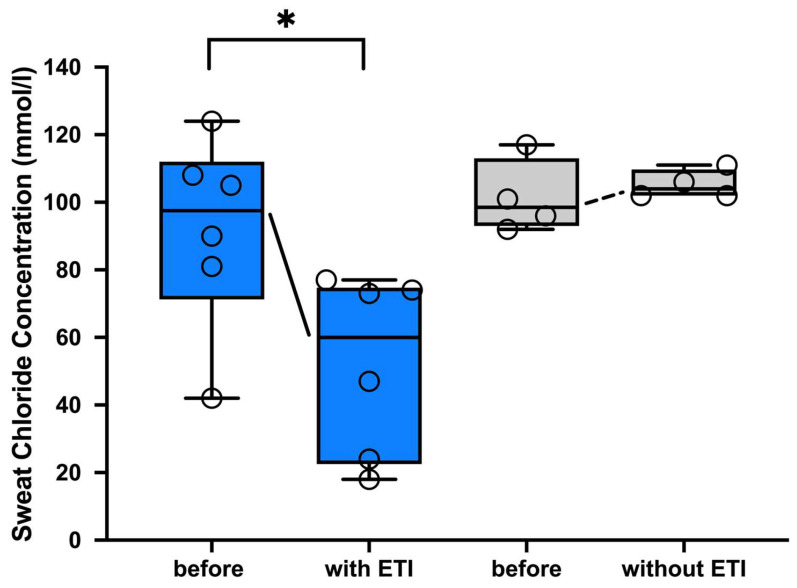
Significant decrease in sweat chloride concentration in people with CF undergoing ETI therapy. Sweat chloride results are shown as individual values (mmol/l) and box-whisker plots with medians, interquartile range, minima, and maxima. Sweat chloride of pwCF (n = 6) is documented immediately before and 1–3 months on ETI therapy compared to sweat chloride of the pwCF control group without ETI (n = 4) at birth and in 2023. Using the Wilcoxon matched-pairs signed rank-test significant values were assessed at *p* < 0.05. Abbreviations: * = *p* < 0.05; ETI= Elexacaftor/Tezacaftor/Ivacaftor; CF= cystic fibrosis; pwCF = people with CF.

After 1–3 months of ETI therapy, sweat chloride significantly decreased by a median of 31.5 mmol/L in all pwCF receiving ETI therapy (range: 24–66 mmol/L) (Figure 1).

As can be seen from the detailed evaluation in Supplementary Table S1, sweat chloride levels decreased more than 30 mmol/L in three pwCF and more than 60 mmol/L in two pwCF after 1–3 months of ETI therapy, with four pwCF showing absolute chloride levels below 60 and two below 30 mmol/L. Only the patient treated with Ivacaftor before starting ETI therapy showed a decrease of only 24 mmol/L. Sweat tests of the control group were also available as part of an internal quality control of a study in 2023. The latter were compared with the sweat test values of the control group, which had been performed after birth at the time of diagnosis. In the control group, only minimal changes in median sweat chloride of +3.5 mmol/L ((−6)–14 mmol/L) were observed over time.

**Figure 2:**
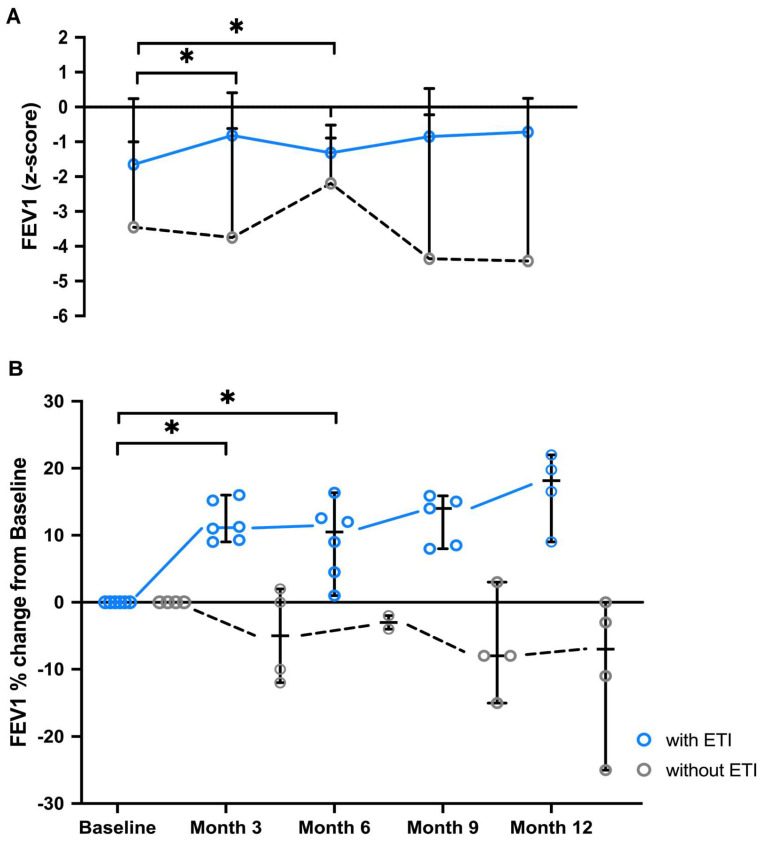
Improvement in FEV_1_ of people with CF on ETI therapy. FEV_1_ of pwCF (n = 6) immediately before and 3/6/9/12 months on ETI therapy compared to the FEV_1_ of the pwCF control group without ETI (n = 4). Significant values with *p* < 0.05 were evaluated using the Friedman test. (**A**) FEV_1_ z-score values are shown as medians with minima and maxima. (**B**) Predicted FEV_1_ values are shown as percentage different from baseline (month 0, immediately before starting ETI therapy). Differences are outlined as individual single values, medians with minima and maxima. Abbreviations: * = *p* < 0.05; ETI = Elexacaftor/Tezacaftor/Ivacaftor; CF = cystic fibrosis; FEV_1_ = forced expiratory volume in 1 s; pwCF = people with CF.

FEV_1_ and FEV_1_/FVC ratios improved in all pwCF who received ETI therapy, as shown in Figure 2 and Supplementary Figure S1. Significant FEV_1_ increases were observed after 3 months (*p* = 0.03) of ETI therapy by a median of 11% (9–15%) and after 6 months (*p* = 0.03) of ETI therapy by 12% (1–16%) compared to baseline (Figure 2A). At the timepoints 9 and 12 months ETI appears to further stabilize the FEV_1_ value, even though changes were not significant compared to baseline. More specifically, at 9 months, the FEV_1_ trended to increase by 14% (8–16%, *p* = 0.06) and at 12 months by 18% (9–22%, *p* = 0.13). In contrast, the FEV_1_ values of the control group trended to deteriorate over time, without significant changes compared to baseline. The median FEV_1_ value of the control group was −4% (2–(−12)%) after 3 months, −3% ((−2)–(−4)%) after 6 months, −8% (3–(−15)%) after 9 months, and −7% (0–(−25)%) after 12 months compared to the baseline value. The different development can also be seen in the *z*-scores (Figure 2A).

VC, which was partially compromised by hyperinflation, tended to increase in pwCF after 3 months under ETI. We could not detect restrictive ventilatory defects, as TLC stayed >80% in all pwCF and showed no significant changes over time, apart from one individual, where TLC temporarily increased above normal ranges, again indicating hyperinflation (Supplementary Figure S1).

**Figure 3:**
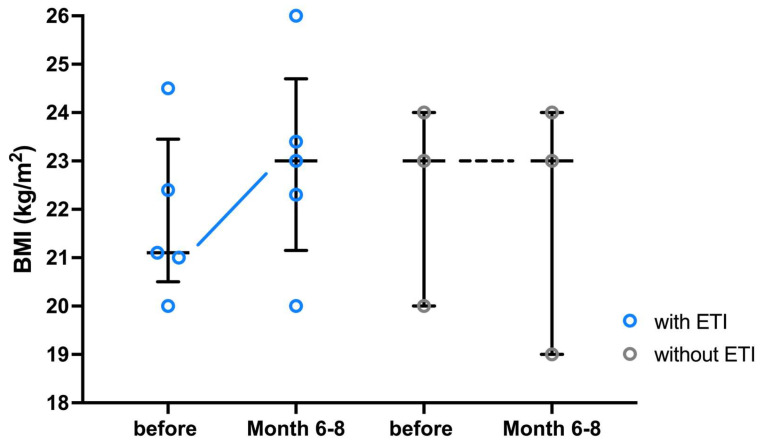
BMI of adult pwCF undergoing ETI therapy. BMI values are presented as individual values and whisker plots with medians and interquartile ranges. BMI values of adult pwCF (n = 5) are shown directly before and after 6–8 months on ETI therapy in comparison to the control group of pwCF without ETI (n = 3). Abbreviations: ETI = Elexacaftor/Tezacaftor/Ivacaftor; BMI = Body-Mass-Index; pwCF = people with CF.

Since most BMI data are only available for month 0 and month 6–8 after the start of ETI therapy, as shown in Supplementary Table S1, the analysis focuses on these two time points. In contrast to the untreated control group, almost all adult pwCF gained weight during ETI therapy (Figure 3). Only in the patient with the CFTR mutations R347P; R1066C, did weight remain constant during ETI therapy. No significant differences in weight gain/BMI before versus on ETI therapy were observed. The CF child who started ETI gained only a small amount of weight during the observation period and remained almost constant at their percentiles after one year of observation. The CF child in the control group remained severely underweight during the observation period, while the BMI percentiles decreased continuously.

After the start of ETI therapy, a mild and transient increase in total bilirubin was observed in four of the six treated pwCF, but this was within 2–21 µmol/L, which the Clinical Chemistry of Ulm University Hospital specifies as reference values for older children and adults, and thus had no clinical consequences (Supplementary Table S1A). During ETI therapy, no adverse events such as rash, intolerance, or psychological abnormalities occurred. In one untreated CF patient, bilirubin levels tended to be higher, but stayed lower than 2× ULN.

### 3.3. Questionnaires

In the retrospective evaluation, apart from the time immediately before the start of ETI therapy, only the questionnaires from 6–8 months after the start of therapy were available. Therefore, the analysis of the questionnaires focused on the following two timepoints: 0 months versus 6–8 months.

**Figure 4:**
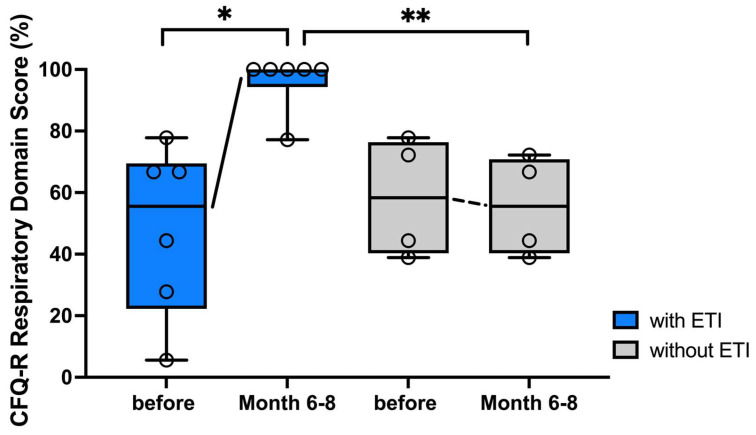
PwCF on ETI therapy report significant improvement in respiratory symptoms compared to baseline. CFQ-R RD results (%) of pwCF (n = 6) immediately before and 6–8 months after initiation of ETI therapy compared to the control group (n = 4) are presented as individual values and box-whisker plots with medians, interquartile range, minima and maxima. Changes after 6–8 months were evaluated using the Wilcoxon matched-pairs signed rank-test, differences to the control group by using the Wilcoxon–Mann–Whitney test. Significant values were assessed at *p* < 0.05. Abbreviations: * = *p* < 0.05, ** = *p* < 0.005; ETI = Elexacaftor/Tezacaftor/Ivacaftor; CFQ-R RD = Cystic Fibrosis Quality of Life Respiratory Domain; pwCF = people with CF.

Respiratory symptoms in the respiratory domain (RD) of the CFQ-R (100% best value) improved significantly by 33% (22–67%) after 6–8 months of ETI therapy compared to the initial value. The median RD percentage of the control group fell from 58% (39–78%) to 56% (39–72%) over the same period (Figure 4). The difference after an observation period of 6–8 months between the control and intervention group is also significant (*p* = 0.005).

As shown in the Supplementary Materials, a more detailed analysis of all 12 CFQ-R domains revealed significant (*p* < 0.05) improvements in additional domains during ETI therapy, such as “physical“, “emotion”, “treatment burden”, and “health perception” (Supplementary Table S3).

**Figure 5:**
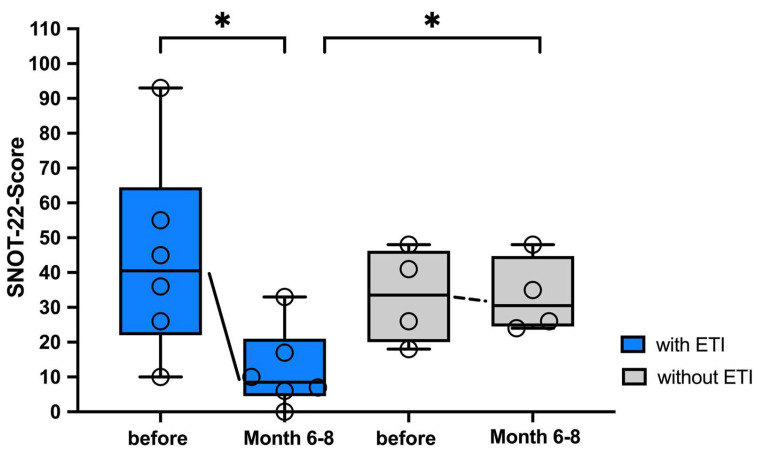
Significant decrease in ENT symptoms of pwCF undergoing ETI therapy compared to baseline. SNOT-22 scores of pwCF (n = 6) are shown immediately before and 6–8 months after the start of ETI therapy compared to the control group (n = 4), presented as individual values and box-whisker plots with medians, interquartile ranges, minima and maxima. Significant reduction in ENT symptoms at 6–8 months on ETI was evaluated using the comparison to the control group using the Wilcoxon–Mann–Whitney test. Significant values were defined by *p* < 0.05. Abbreviations: * = *p* < 0.05; ETI = Elexacaftor/Tezacaftor/Ivacaftor; ENT = ear, nose and throat; SNOT-22 = Sino-Nasal Outcome Test-22; pwCF = people with CF.

With ETI therapy, ENT symptoms were significantly reduced compared to the control group and compared to baseline (Figure 5). Since higher values in the SNOT-22 are associated with more severe ENT symptoms, a decrease in the SNOT score indicates a reduction in ENT symptoms. The median SNOT score (worst value 110) decreased from 40.5 (10–93) to 8.5 (0–33) after 6–8 months of ETI treatment. The control group started with an initial SNOT score of 33.5 (18–48) points and deteriorated to 43.5 (24–48) points over the same period.

**Figure 6:**
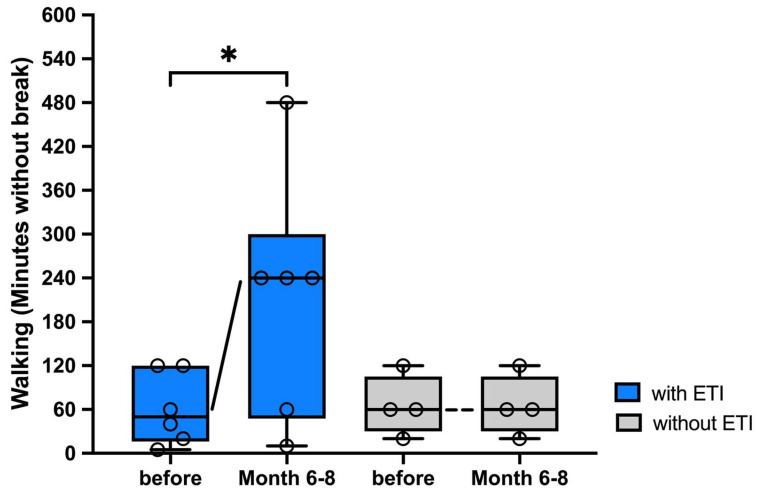
Significant improvement in physical performance of pwCF after 6–8 months on ETI therapy compared to baseline. Shown are the walking minutes pwCF could complete immediately before and 6–8 months after initiation of ETI therapy (n = 6) compared to the control group (n = 4). The number of minutes is presented as individual values and box-whisker plots with medians, interquartile ranges, minima, and maxima. Significant values were defined by *p* < 0.05 by using the Wilcoxon matched-pairs signed rank-test. Abbreviations: * = *p* < 0.05; ETI= Elexacaftor/Tezacaftor/Ivacaftor; pwCF = people with CF.

In pwCF undergoing ETI therapy, a significant increase in the number of walking minutes without a break was documented for a median of 150 min (5–360 min). In contrast, the physical performance measured by walking did not change at all in all the pwCF in the control group after an observation period of 6–8 months (Figure 6). Three pwCF of the intervention group had a pedometer and confirmed improved activity after 6–8 months on ETI therapy with a median increase of 3744 steps (960–5000 steps) per day. Treated pwCF also reported a median increase in cycling minutes by 30 min (0–60 min) and an improvement in stair climbing by two floors (2–17 floors) in pwCF after 6–8 months on ETI.

The evaluation of the TSQM revealed a consistent medication satisfaction of almost always 100% in all four domains of “effectiveness”, “side effects”, “convenience”, and “overall satisfaction”, as can be seen from the detailed presentation of all data in the appendix (Supplementary Table S2).

## 4. Discussion

In this study, the real-world effectiveness of ETI was evaluated in a small cohort of pwCF with non-phe508del responsive CFTR mutations. Despite the small sample size and heterogeneity of the pwCF in our cohort, we conducted a systematic data collection and analysis comparable to clinical study standards. The pwCF in our cohort share the following two key features: they have CFTR mutations that unequivocally cause CF; however, in the treated group, at least one mutation permits the production of some CFTR protein. Instead of comparing patients, in our study, we focused on evaluating individual patient trajectories before and during ETI treatment over a 12-month observation period, using both clinical and objective parameters. Importantly, we limited our study to patients with mutations recently confirmed as responsive to ETI in vitro. Within this case series, we observed a measurable positive effect of ETI therapy, including reductions in sweat chloride levels—a key objective parameter. In four treated pwCF, sweat chloride levels decreased to <60 mmol/L, and reached normal levels (<30 mmol/L) in two of these cases. With reference to recently published case reports from the FCUP, reported by Burgel et al. [23], the decision to continue ETI was based on reduction in sweat chloride of at least 20 mmol/l and/or FEV_1_ improvement of at least 10%. Site-specific abilities of our study include ENT evaluation and the assessment of physical activity and quality of life through questionnaires—parameters not routinely evaluated in CF centers. Notably, a sustained positive effect of ETI was observed over one year—in contrast to the 4–6 weeks previously reported for another cohort of non-phe508del pwCF [23]. The absence of adverse events and consistent satisfaction reported in the TSQM suggest good tolerability of ETI in this group.

The pwCF with R347P/R1066C was included in the evaluation at time points 0, 3, and 6 months, but her data were excluded from time points 9 and 12 months, during which she received a proteasome inhibitor (in addition to ETI) to treat a (semi)malignant disease, a monoclonal gammopathy of renal significance, developed from a longer persistent monoclonal gammopathy of unclear significance (MGUS). Since she benefited regarding CF symptoms, she wished to continue ETI and is now in a stable condition. So far, ETI by itself has not been described to cause malignancies, making a causal relationship unlikely.

The FCUP evaluated the ETI effects in pwCF over 12 years of age with FDA-approved non-phe508del CFTR mutations. Despite the relatively large cohort of 45 responders and 39 non-responders, mutation heterogeneity precluded subgroup analyses, except for N1303K, with six homozygous cases. Over a 4–6-week period, a median sweat chloride decrease of −36 mmol/L and a median FEV_1_ increase of 12% was documented [23]. Similarly, our ETI-treated cohort showed a median sweat chloride decrease of −31.5 mmol/L and an FEV_1_ increase of 11% after three months. Additionally, we observed FEV_1_ stabilization over 12 months, with a median increase of 18% compared to baseline, narrowly missing significance due to one dropout. In contrast to the FCUP, which showed limited effects in patients switched from Ivacaftor to ETI, the single individual in our cohort demonstrated slight improvements, including an 8–9% increase in FEV_1_, BMI increase from 25 to 27 kg/m^2^, and a sweat chloride decrease of −22 mmol/L.

In comparison to ETI studies in pwCF with at least one phe508del mutation, clinical outcomes in our cohort were similar. Reported FEV_1_ increases of 11–14% and BMI gains of +1 kg/m^2^ after six months align with our findings. However, the reduction in sweat chloride (−41 to −46 mmol/L) was slightly greater than in our cohort, likely due to our earlier measurement (1–3 months) [6,10].

It must be pointed out that pwCF with non-phe508del mutations are very rare so that studies with larger case numbers with identical combinations of mutation variants are unrealistic to achieve. The cohort size reflects the rarity of the mutations studied; among approximately 300 pwCF treated at the two CF centers involved, the prevalence of such mutations is exceedingly low. Larger studies would be logistically unfeasible within a manageable timeframe. A search of the CFTR2 database (cftr2.org), which includes nearly 40,000 CF patients, underscores this challenge. For instance, only two patients share the mutation combination R347P;R1066C, none the combination Q220X;M1101K, and very few other combinations, such as 2789+5G->A;M1101K (three patients), G542X;M1101K (three patients), CFTRdele2,3;M1101K (two patients), and G551D;M1101K (six patients) [31].

In Germany, more than 80% of pwCF receive ETI therapy, so that only very few pwCF are available as controls, including those with CFTR mutations that do not allow CFTR protein production. Hence, the group of untreated pwCF can only serve as an indication that the expected course of pwCF without ETI may also be observed in our clinical setting, where all pwCF have comparable access to common CF treatment, no matter what the underlying CFTR mutations are. Nevertheless, our results suggest that our methods of evaluation were carried out correctly, e.g., that in the untreated group, the objective parameters of sweat chloride stayed high and the lung function or BMI remained unchanged over the observation period. Since the focus of this brief medical report was the course of disease, and to ensure that our observations were reliable, we chose a rather long period of 12 months. The pwCF in our cohort thus have in common that they exhibit CFTR mutations, which distinctly cause CF including the classical clinical CF phenotype.

We are convinced that it is in the best interests of pwCF with rare mutations to publish real world data, not only larger studies driven mainly by the interests of pharmaceutical companies. Further, we advocate for the use of in vitro data to justify ETI treatment in rare cases and have based our approach on in vitro findings [20] demonstrating the responsiveness of the CFTR mutations M1101K and R347P, recently corroborated by others [31].

This study is the first to analyze a cohort of pwCF with rare non-phe508del CFTR mutations using parameters such as the SNOT-22 score, CFQ-R, and AQ. Comparisons with studies involving at least one phe508del mutation reveal consistent findings. For instance, the pivotal ETI study [6] reported a 20% improvement in the CFQ-R RD domain after six months, whereas our cohort showed a 33% improvement after 6–8 months. Similarly, ENT symptom improvements described by Bode et al. [15] (17-point SNOT score improvement after nine months) align with our 28-point improvement after 6–8 months. Although data on physical activity parameters in ETI therapy are scarce, pilot studies using spiroergometry confirm our findings of improved fitness [32,33].

Importantly, this study is the first to provide data on a child with CF under 12 years of age with a non-phe508del mutation. The child’s outcomes—including improvements in FEV_1_, sweat chloride, and CFQ-R—mirrored those of children with at least one phe508del mutation [34].

The major strength of this study lies in its long-term analysis of pwCF with rare non-phe508del CFTR variants, for which data on ETI effects are limited. Conducted over 12 months—exceeding most published durations [23]—this study evaluated not only lung function and BMI, but also ENT symptoms and daily activity levels. Furthermore, as this study was conducted without industrial sponsorship, it provides independent results. To our knowledge, our study is the first to report changes in ENT symptoms and activity with ETI therapy in pwCF with non-phe508del CFTR mutations. This is also the first report of ENT symptom and activity changes with ETI therapy in pwCF with non-phe508del mutations. Although these mutations are rare, our cohort was evenly balanced between sexes, and the inclusion of one child in each group enhanced comparability. Five pwCF in our ETI cohort carry the M1101K mutation, historically defined as a minimal function mutation due to <10% chloride transport compared to wild-type CFTR in vitro. Early findings that Ivacaftor or Ivacaftor/Tezacaftor alone did not significantly increase CFTR function [6] have since been revised, with ETI being shown to achieve >10% chloride transport. These results, which prompted an extension of the FDA approval in the US and UK in 2020 [20], were recently confirmed by Biehler et al. [31].

The major limitation of this study is the small cohort size, reflecting the rarity of non-phe508del mutations, which limits statistical power. Similarly, the control group was small, heterogeneous, and not age matched, due to widespread access to ETI in industrialized countries. All statistical analyses should therefore be interpreted with caution and cannot be applied to the total population of pwCF. Moreover, we cannot rule out observer and patient bias, even though we tried to ameliorate this by an extended observation period. While larger studies would be ideal, they are unrealistic given the low incidence of these mutations. Reliance on in vitro data to predict clinical benefit remains essential for offering ETI to such patients. Personalized in vitro testing of patient-derived respiratory epithelia may provide additional insights, although such tests are not yet part of routine diagnostics [35]. Combining these approaches with stringent monitoring could justify treatment based on in vitro findings. We advocate for changes in the EU’s regulatory approach for rare diseases to better support such cases.

## 5. Conclusions

In the series of six cases of pwCF reported here, we could observe detectable positive effects of the CFTR modulator ETI, which resembled reports on pwCF with at least one phe508del mutation and included the objective parameter sweat chloride as well as clinical parameters. The comparatively long observation period enhances credibility and robustness of the beneficial clinical effects. However, this reported small number of cases does not allow a definite conclusion on any given genotype. Despite the low number of cases and heterogeneity of our cohort, we have attempted to carry out a comparable data collection and analysis as required in clinical studies. The cohort size reflects the rarity of the mutations studied; among approximately 300 pwCF treated at the two CF centers involved, the prevalence of such mutations is exceedingly low. Five ETI-treated pwCF in our cohort were heterozygous for the CFTR mutations M1101K and one for R347P, previously shown to respond to ETI in vitro. Based on our results and comparable studies, we advocate for approval of ETI in the EU for pwCF with non-phe508del CFTR mutations based on in vitro responsiveness. Additional clinical evidence is necessary and may be supplemented by personalized in vitro studies, e.g., with patient-derived epithelial cells.

## Figures and Tables

**Table 1:** Demographic characteristics of the intervention group (patients 1–6) and the control group (patients A–D) including age, gender, CFTR mutations, and CFTR modulator premedication are summarized.

Patient	Age (y)	Gender	CFTR-Mutation 1	CFTR-Mutation 2	ETI	Ivacaftor Before ETI
1	45	f	R347P *	R1066C	Yes	No
2	6	m	Q220X	M1101K *	Yes	No
3	66	f	2789+5G>A	M1101K *	Yes	No
4	35	m	G542X	M1101K *	Yes	No
5	45	m	CFTRdele2,3	M1101K *	Yes	No
6	29	f	G551D *	M1101K *	Yes	Yes
A	22	f	Q39X	CFTRdele17a,17b	No	No
B	7	m	Q39X	R785X	No	No
C	46	f	phe508del *	phe508del *	No	No
D	23	f	I506S	CFTRdele14b-17b	No	No

* FDA-approved. Abbreviations: y = year; f = female; m = male; ETI= Elexacaftor/Tezacaftor/Ivacaftor; CFTR = Cystic Fibrosis Transmembrane Conductance Regulator; FDA = Food and Drug Administration.

## Data Availability

The data presented in this study are available on request from the corresponding author and are not yet openly available due to ongoing analysis and observation.

## Supplementary Materials

The following supporting information can be downloaded at: https://www.mdpi.com/article/10.3390/arm92060049/s1, Table S1: detailed evaluation of the clinical data; Table S2: detailed evaluation of the questionnaires; Table S3: detailed CFQ-R Evaluation.
