# Supplementary material for: Successful Therapy over 12 Months of People with Cystic Fibrosis with Rare Non-phe508del Cystic Fibrosis Transmembrane Conductance Regulator (CFTR) Mutations with Elexacaftor/Tezacaftor/Ivacaftor (ETI)
Source: Adv Respir Med. 2024 Dec 20;92(6):559–72. doi: 10.3390/arm92060049 (PMC11672886; doi:10.3390/arm92060049)
Supplement: Supplementary file 1 [file arm-92-00049-s001.zip › arm-3269476-supplementary.pdf]

Table S1: Detailed evaluation of the clinical data

S1A

Patient	CFTR-Mutation 1	CFTR-Mutation 2	ETI	ST (mmol/L)		Percentage Predicted FEV1					BMI (kg/m²)					Bilirubin (µmol/l)				
				Before	After	M0	M3	M6	M9	M12	M0	M3	M6	M9	M12	M0	M3	M6	M9	M12
1	R347P*	R1066C	Yes	90	24	35	46	47	n.d.	46	21	22	23	n.d.	23	10	25	9	17	18
2	Q220X	M1101K*	Yes	124	55	81	90	82	89	103	kid	kid	kid	kid	kid	4	5	4	4	5
3	M1101K*	2789+5G>A	Yes	108	62	71	87	80	85	80	20	20	20	20	20	3	7	10	15	13
4	G542X	M1101K*	Yes	105	73	81	93	94	97	98	22	22	23	23	23	8	14	10	15	13
5	CFTRdele2,3	M1101K*	Yes	81	47	53	68	69	68	73	21	21	22	22	22	10	14	14	14	17
6	G551D*	M1101K*	Yes	42	18	83	92	87	91	n.d.	25	26	26	27	n.d.	15	12	10	7	n.d.
7	1518C>G	R851X	Yes	109	84	96	107	107	n.d.	n.d.	21,1	19,9	20,4	n.d.	n.d.	5,1	10,2	11,9	n.d.	n.d.
8	R1066C	R1066C	Yes	96	38	88	111	n.d.	n.d.	n.d.	17	16,3	n.d.	n.d.	n.d.	<3,4	6,8	n.d.	n.d.	n.d.
9	N1303K	N1303K	Yes	95	87	44	97	102	104	102	17,5	19,6	20,4	21	22,8	13,6	17	22,1	20,4	n.d.
A	Q39X	CFTRdele17a,17b	No	92	106	103	105	n.d.	106	100	23	24	24	n.d.	24	n.d.	n.d.	n.d.	5	5
B	Q39X	R785X	No	117	111	61	51	57	46	36	kid	kid	kid	kid	kid	13	n.d.	6	6	8
C	phe508del*	phe508del*	No	96	102	50	50	48	42	50	24	23	23	24	24	3	4	n.d.	4	n.d.
D	I506S	CFTRdele14b-17b	No	92	106	47	35	n.d.	39	36	20	n.d.	19	n.d.	20	22	22	19	30	15

*FDA-approved

S1B

Patient	CFTR-Mutation 1	CFTR-Mutation 2	ETI	CRP (mg/l)					Leucocytes (Giga/L)					AST (U/L)					ALT (U/L)					GGT (U/L)				
				M0	M3	M6	M9	M12	M0	M3	M6	M9	M12	M0	M3	M6	M9	M12	M0	M3	M6	M9	M12	M0	M3	M6	M9	M12
1	R347P*	R1066C	Yes	n.d.	3,7	15,2	36,5	11,8	14,7	13	5	11,1	8,8	24	26	41	25	18	7	11	63	43	19	60	37	84	64	56
2	Q220X	M1101K*	Yes	0,8	n.d.	n.d.	n.d.	<0,6	11,7	n.d.	7,7	4	5	30	32	28	28	27	6	11	8	10	10	12	12	7	13	13
3	M1101K*	2789+5G>A	Yes	n.d.	n.d.	<0,6	n.d.	n.d.	6,4	7,2	5	n.d.	5,1	23	24	23	23	23	11	17	25	6	17	11	13	13	13	11
4	G542X	M1101K*	Yes	1,21	0,17	<0,1	<0,1	0,17	9,4	5,5	6,7	6	6,1	29	42	35	47	37	30	70	69	93	65	14	18	18	16	20
5	CFTRdele2,3	M1101K*	Yes	<0,1	4,51	<0,1	<0,1	<0,1	7,2	6	5,7	6	5,2	35	31	50	52	45	26	35	46	50	48	15	53	13	15	18
6	G551D*	M1101K*	Yes	0,83	<0,1	1	4	n.d.	6,9	5,6	9,64	3,92	n.d.	21	21	19	20	n.d.	8	8	5	6	n.d.	10	12	8	16	n.d.
7	1518C>G	R851X	Yes	n.d.	n.d.	n.d.	n.d.	n.d.	7,09	n.d.	6,2	n.d.	n.d.	15	29	18	n.d.	n.d.	12	18	13	n.d.	n.d.	15	13	16	n.d.	n.d.
8	R1066C	R1066C	Yes	n.d.	n.d.	n.d.	n.d.	n.d.	6,57	n.d.	n.d.	n.d.	n.d.	20	39	n.d.	n.d.	n.d.	31	47	n.d.	n.d.	n.d.	12	12	n.d.	n.d.	n.d.
9	N1303K	N1303K	Yes	1,18	n.d.	n.d.	2,05	n.d.	7,24	9,06	n.d.	7,78	n.d.	20	24	19	19	n.d.	9	15	13	15	n.d.	11	10	9	11	n.d.
A	Q39X	CFTRdele17a,17b	No	2,3	1,2	n.d.	n.d.	2,2	7,8	n.d.	n.d.	n.d.	11,6	24	n.d.	n.d.	n.d.	21	7	n.d.	n.d.	n.d.	12	15	n.d.	n.d.	n.d.	17
B	Q39X	R785X	No	<0,6	n.d.	<0,6	3,9	13,5	6,6	n.d.	6,9	11,4	15,7	29	n.d.	35	24	24	15	n.d.	24	5	9	n.d.	n.d.	13	n.d.	n.d.
C	phe508del*	phe508del*	No	2,7	3,2	n.d.	10,4	10,3	7,1	8,7	n.d.	9,8	n.d.	26	27	n.d.	25	n.d.	22	22	n.d.	24	n.d.	61	79	n.d.	78	n.d.
D	I506S	CFTRdele14b-17b	No	4,6	n.d.	n.d.	5,3	9,1	3,3	5	n.d.	3,3	3,1	24	20	29	27	17	9	<5	7	7	7	69	39	49	48	35

*FDA-approved

S1C

Patient	CFTR-Mutation 1	CFTR-Mutation 2	ETI	Percentage Predicted TLC					Percentage Predicted VC					FEV1/FVC				
				M0	M3	M6	M9	M12	M0	M3	M6	M9	M12	M0	M3	M6	M9	M12
1	R347P*	R1066C	Yes	85	84	83	n.d.	n.d.	50	60	55	n.d.	n.d.	0.66	0.70	0.73	n.d.	n.d.
2	Q220X	M1101K*	Yes	105	107	100	101	111	92	94	94	91	94	0.77	0.84	0.77	0.86	0.95
3	M1101K*	2789+5G>A	Yes	123	147	152	136	133	89	107	94	101	95	0.62	0.63	0.67	0.65	0.65
4	G542X	M1101K*	Yes	110	109	102	113	110	91	102	91	102	107	0.89	0.90	0.92	0.95	0.91
5	CFTRdele2.3	M1101K*	Yes	108	107	107	104	107	78	89	91	91	95	0.67	0.76	0.76	0.75	0.76
6	G551D*	M1101K*	Yes	96	97	103	100	n.d.	85	97	98	95	n.d.	0.97	0.94	0.94	0.94	n.d.
A	Q39X	dele17a.17b	No	124	122	n.d.	124	142	115	119	n.d.	117	112	0.77	0.79	n.d.	0.79	0.77
B	Q39X	R785X	No	147	152	125	124	149	82	76	70	52	37	0.66	0.60	0.72	0.78	0.85
C	phe508del*	phe508del*	No	113	126	130	120	113	86	69	72	82	87	0.52	0.58	0.54	0.46	0.52
D	I506S	dele14b-17b	No	85	n.d.	n.d.	85	80	58	48	n.d.	56	56	0.71	0.72	n.d.	0.61	0.57

*FDA-approved

Table S1: Individual data of CFTR mutations of study participants and clinical parameters over time. Values of people with CF (pwCF) on ETI who were excluded due to exclusion criteria are marked in grey. **S1A:** Sweat chloride concentrations of pwCF before and 1-3 months after initiation of ETI therapy (patient 1-6), and an internal quality control at birth and in 2023 (patient A-D). Development of FEV_1 (percentage predicted), BMI (kg/m²) and bilirubin (µmol/L) at time points 0, 3, 6, 9 and 12 months. **S1B:** Values of CRP (mg/dl), Leucocytes (Giga/l), GOT, GPT and GGT (U/L) of pwCF at time points 0, 3, 6, 9 and 12 months. **S1C:** Additional lung function parameters to characterize obstructive (FEV_1/FVC) versus restrictive changes (TLC, VC) at the time points 0/3/6/9/12 months.

Abbreviations: CF= Cystic Fibrosis, M= Month, CFTR= Cystic Fibrosis Transmembrane Conductance Regulator, ETI= Elexacaftor/Tezacaftor/Ivacaftor, FDA= Food and Drug Administration, ST= Sweat Chloride Test, FEV1= Forced Expiratory Volume in 1 second, BMI= Body-Mass-Index, CRP= C-reactive proteine, ALT= Alanine aminotransferase, AST= Aspartate aminotransferase, GGT=Gamma glutamyl transferase, TLC= Total Lung Capacity, VC= Vital Capacity, FVC= Forced Expiratory Volume; n.d. = not determined

Table S2: Detailed evaluation of the questionnaires

Patient	CFTR-Mutation 1	CFTR-Mutation 2	ETI	CFQ-R RD		SNOT-22-Score		Activity (Walking)		TSQM							
										Evectiveness		Side Effects		Convenience		Global Satisfaction	
				M0	M 6-8	M0	M 6-8	M0	M 6-8	M 1	M 6-8	M 1	M 6-8	M 1	M 6-8	M 1	M 6-8
1	R347P*	R1066C	Yes	6%	77%	93	33	5 min.	10 min.	100	100	100	100	100	100	100	100
2	Q220X	M1101K*	Yes	67%	100%	10	0	40 min.	60 min.	100	100	100	100	94	94	100	100
3	M1101K*	2789+5G>A	Yes	78%	100%	36	17	60 min.	240 min.	100	100	100	100	100	100	100	100
4	G542X	M1101K*	Yes	67%	100%	26	10	120 min.	480 min.	100	100	100	100	100	100	100	100
5	CFTRdele2,3	M1101K*	Yes	44%	100%	45	6	120 min.	240 min.	100	100	100	100	89	94	100	100
6	G551D*	M1101K*	Yes	28%	100%	55	7	20 min.	240 min.	100	100	100	100	78	100	93	100
A	Q39X	CFTRdele17a,17b	No	39%	39%	48	48	120 min.	120 min.	n.d.	n.d.	n.d.	n.d.	n.d.	n.d.	n.d.	n.d.
B	Q39X	R785X	No	44%	44%	26	26	20 min.	20 min.	n.d.	n.d.	n.d.	n.d.	n.d.	n.d.	n.d.	n.d.
C	phe508del*	phe508del*	No	72%	72%	41	35	60 min.	60 min.	n.d.	n.d.	n.d.	n.d.	n.d.	n.d.	n.d.	n.d.
D	I506S	CFTRdele14b-17b	No	78%	67%	18	24	60 min.	60 min.	n.d.	n.d.	n.d.	n.d.	n.d.	n.d.	n.d.	n.d.

*FDA-approved

Table S2: CFTR mutations of study participants and individual data of the questionnaires over time. Development of CFQ-R RD, SNOT-22-Score and activity in walking minutes without a break of pwCF at the timepoints 0 and 6-8 months. TSQM Evaluation of pwCF under ETI therapy (patient 1-6) at the time points 1 and 6-8 months after start of therapy.

Abbreviations: CF= Cystic Fibrosis, M= Month, CFTR= Cystic Fibrosis Transmembrane Conductance Regulator, ETI= Elexacaftor/Tezacaftor/Ivacaftor, FDA= Food and Drug Administration, TSQM= Treatment Satisfaction Questionnaire for Medication, CFQ-R RD= Cystic Fibrosis Quality of Life Respiratory Domain, SNOT-22 = Sino-Nasal Outcome Test-22, TSQM= Treatment Satisfaction Questionnaire for Medication, n.d. = not determined

Table S3: Detailed CFQ-R Evaluation

CFQ-R-Domains	ETI Group M [Min; Max]		Countrol Group M [Min; Max]		*P*-value ETI/C	*P*-value ETI/ETI
	Before	Month 6-8	Before	Month 6-8	Month 6-8	Before/Month 6-8
Physical	67% [4; 93]	98% [42; 100]	77% [50; 96]	73% [62; 96]	0.16	**0.03***
Vitality	50% [0; 80]	81% [58; 93]	58% [25; 67]	58% [25; 67]	**0.04***	0.09
Emotion	73% [20; 93]	93% [27; 100]	63% [13; 73]	67% [13; 80]	0.067	**0.03***
Social	56% [39; 89]	93% [27; 100]	56% [39; 83]	67% [39;78]	0.3	0.13
Role	83% [11; 100]	96% [44; 100]	82% [50; 100]	75% [50; 100]	0.33	0.06
Body Image	72% [44; 100]	94% [78; 100]	39% [11; 78]	50% [11; 78]	**0.02***	0.13
Eating	100% [67; 100]	100% [89; 100]	67% [0; 100]	67% [0; 100]	0.3	0.5
Treatment Burden	56% [4; 67]	94% [67; 100]	67% [44; 89]	67% [44; 89]	0.08	**0.03***
Health Perceptions	56% [0; 67]	100% [11; 100]	33% [33; 56]	45% [33; 67]	0.08	**0.03***
Respiratory	56% [6; 78]	100% [78; 100]	58% [39; 78)	56% [39; 72]	**0.005****	**0.03***
Digest	72% [56; 100]	83% [78; 100]	89% [78; 100]	83% [78; 100]	>0.99	0.5
Weight	67% [0; 100]	100% [33; 100]	33% [0; 100]	50% [0; 100]	0.45	0.5

Table S3: Significant improvement in CFQ-R-Domains Respiratory Symptoms, Vitality and Body Image of pwCF undergoing ETI therapy compared to the control group at 6-8 months after the start of therapy using the Wilcoxon matched-pairs signed rank-test. Significant improvement in CFQ-R-Domains Physical, Emotion, Treatment Burden, Health Perceptions and Respiratory Symptoms of CF people after 6-8 months of ETI therapy compared to baseline using the Wilcoxon matched-pairs signed rank-test. CFQ-R values in % shown as medians (M), minima (Min) and maxima (Max). The twelve CFQ domains of pwCF under ETI therapy (n=6) at the time immediately before and 6-8 months after the start of ETI therapy compared to the control group without ETI therapy (n=4).

*Abbreviations: *=p<0.05, **=p>0.005, pwCF= people with Cystic Fibrosis, ETI= Elexacaftor/Tezacaftor/Ivacaftor, CFQ-R= Cystic Fibrosis Quality of Life*

Supplementary Figure S1:

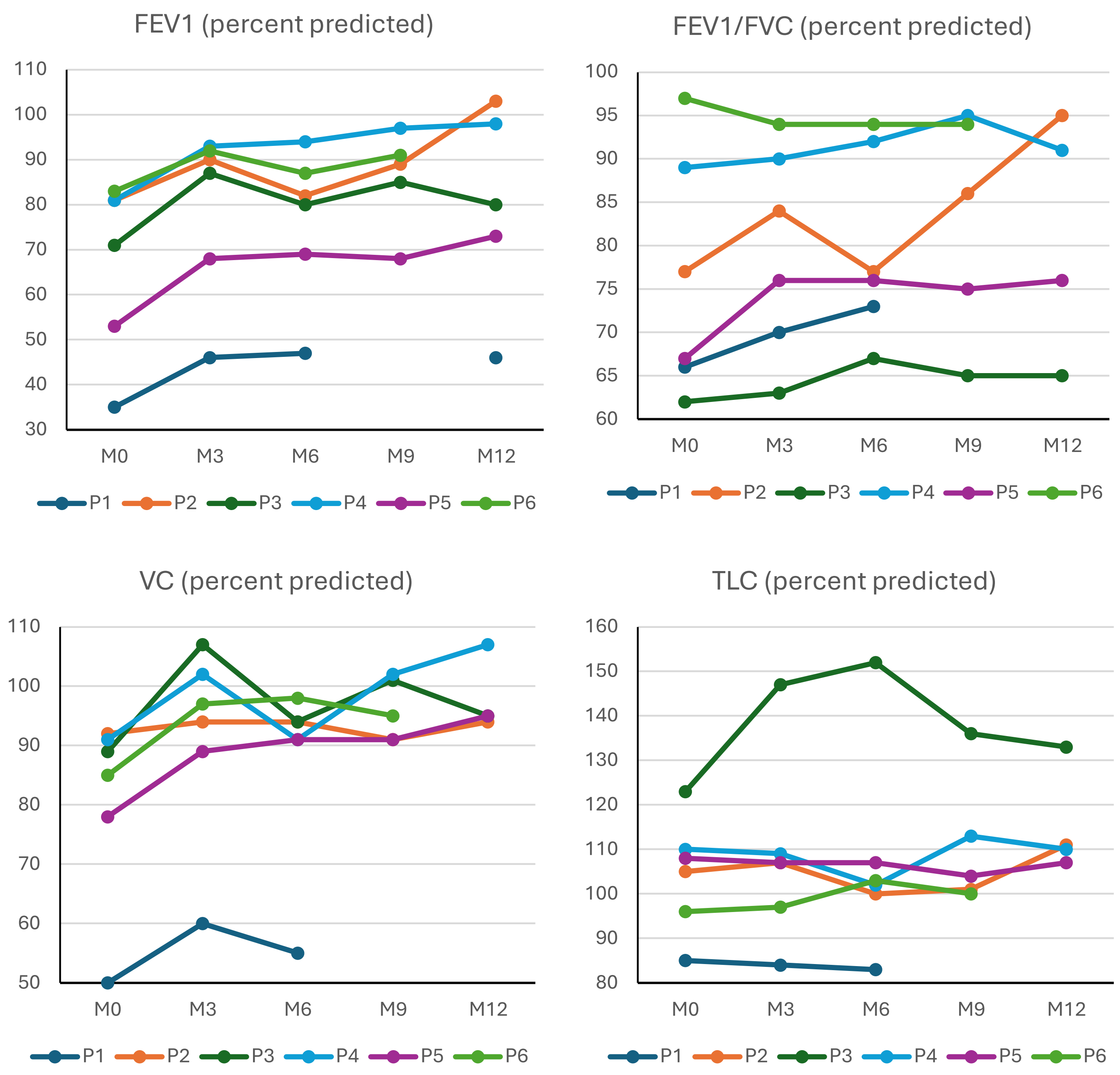

Figure S1: Lung function parameters of ETI-treated pwCF. Individual development of lung function in ETI-treated pwCF showing obstructive (FEV_1, FEV_1/FVC) versus restrictive changes (TLC, VC) at time points 0, 3, 6, 9 and 12 months. Pulmonary function testing was performed according to Global Lung Function Initiative/European Respiratory Society guidelines. Results are shown as percentage of the respective predicted values. To reflect the best maneuver the patient was able to perform, the higher value between FVC and VCin was recorded and used for VC analysis.

Abbreviations: M= Month, pwCF= people with Cystic Fibrosis, FEV_1*= Forced Expiratory Volume in 1 second, FVC= Forced Vital Capacity, VC= Vital capacity, TLC= Total Lung Capacity, VCin= Inspiratory Vital Capacity.*
